# Supplementary material for: Characteristics of Streptococcus agalactiae Colonizing Nonpregnant Adults Support the Opportunistic Nature of Invasive Infections
Source: Microbiol Spectr. 2022 May 23;10(3):e01082-22. doi: 10.1128/spectrum.01082-22 (PMC9241740; doi:10.1128/spectrum.01082-22)
Supplement: SUPPLEMENTAL FILE 1 — Supplemental material. Download spectrum.01082-22-s001.pdf, PDF file, 0.1 MB [file spectrum.01082-22-s001.pdf]

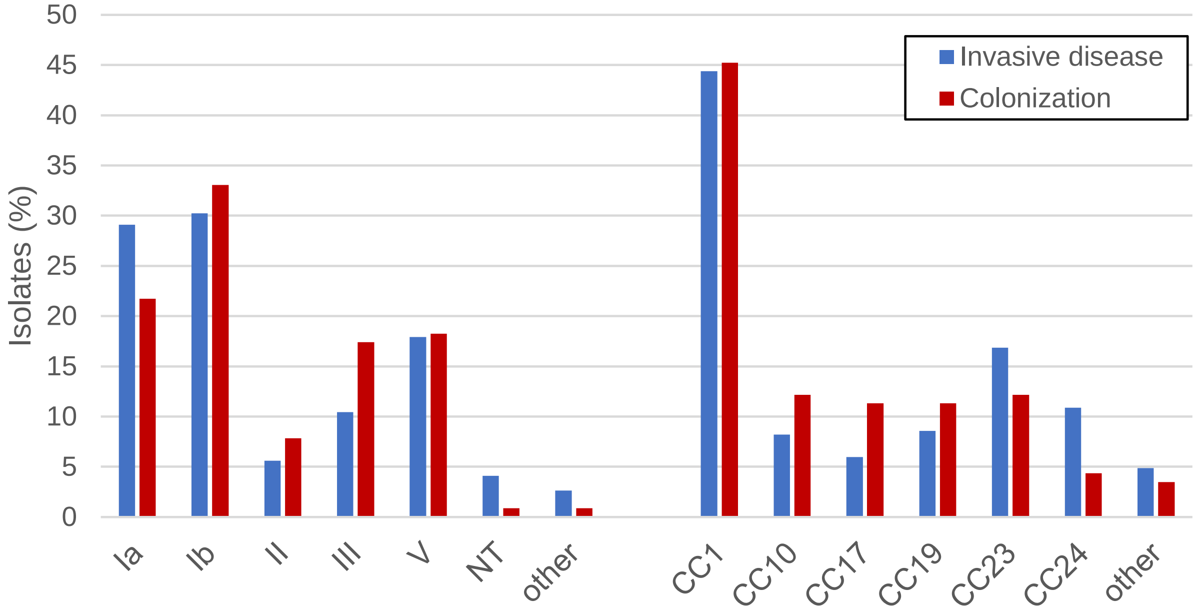

Figure S1. Comparison of serotype and clonal complex distribution of GBS colonizing and causing invasive disease in non-pregnant adults in Portugal (2013-2015). Other serotypes are IV, VI and IX. Other CCs are CC4, CC7, CC22, CC26, CC103 and CC130.
